# The association between sedentary behavior and MASLD in overweight and obese adults: investigating the role of inflammatory markers using NHANES data (2017–March 2020)

**DOI:** 10.3389/fnut.2025.1579453

**Published:** 2025-06-27

**Authors:** Zehong Zhou, Linfang Li, Chusi Wang, Shiqi Li, Pengfei Chen, Jiesheng Huang, Ming Peng

**Affiliations:** ^1^Department of Clinical Laboratory Medicine, Zhongshan Hospital of Traditional Chinese Medicine, Zhongshan, China; ^2^Department of Clinical Laboratory Medicine, Sun Yat-sen University Cancer Center, State Key Laboratory of Oncology in South China, Guangdong Provincial Clinical Research Center for Cancer, Guangzhou, China; ^3^Department of Hepatobiliary Surgery, The Third Affiliated Hospital of Sun Yat-sen University, Guangzhou, China; ^4^Department of Classical Chinese Medicine, Zhongshan Hospital of Traditional Chinese Medicine, Zhongshan, China

**Keywords:** MASLD, metabolic dysfunction associated steatotic liver disease, sedentary behavior (SB), inflammatory markers, NHANES (National Health and Nutrition Examination Survey), mediation analysis

## Abstract

**Background:**

Metabolic dysfunction-associated steatotic liver disease (MASLD) has been linked to sedentary behavior (SB), yet the extent to which systemic inflammation mediates this relationship remains unclear. This study aims to demonstrate the mediating function of inflammatory markers in the link between sedentary behavior and metabolic dysfunction-associated steatotic liver disease (MASLD) in overweight and obese individuals.

**Methods:**

In this cross-sectional study, we analyzed pre-pandemic data (2017–March 2020) from the National Health and Nutrition Examination Survey (NHANES), including 3,729 overweight/obese adults with MASLD defined by a controlled attenuation parameter (CAP) ≥ 302 dB/m. Self-reported SB (≥480 min/day vs. <480 min/day) and vigorous recreational activity were assessed alongside inflammatory markers (high-sensitivity C-reactive protein [HSCRP], albumin [ALB], white blood cell count [WBC], and neutrophil count [NE]). And weighted multivariable logistic and linear regression models, as well as mediation analyses, were conducted to account for the complex sampling design of the NHANES data.

**Results:**

Weighted logistic regression showed that severe SB was associated with higher MASLD odds (OR = 1.43, 95% CI: 1.02–1.99), which further increased (OR = 2.88, 95% CI: 1.77–4.71) in participants lacking vigorous physical activity. Independent predictors of MASLD included lower ALB (OR = 0.55) and higher WBC (OR = 1.16) and NE (OR = 1.17). Mediation analysis indicated that HSCRP, ALB, WBC, and NE, respectively, accounted for 10.48, 3.23, 7.17, and 6.46% of the SB – MASLD association.

**Conclusion:**

Our findings suggest that severe sedentary behavior is an independent risk factor for MASLD, with some evidence suggesting that this relationship may be influenced by inflammatory markers. However, longitudinal studies are necessary to better understand the nature of these associations and to explore the underlying mechanisms involved.

## 1 Introduction

In June 2023, non-alcoholic fatty liver disease (NAFLD) and metabolic associated fatty liver disease (MAFLD) were renamed metabolic dysfunction associated steatotic liver disease (MASLD) to emphasize the role of metabolic dysfunction in its development (1). Its prevalence has risen globally, with overweight and obese populations disproportionately affected, showing rates approaching 70% (2). Beyond simple steatosis, MASLD encompasses a spectrum ranging from non-alcoholic steatohepatitis (NASH) to cirrhosis and hepatocellular carcinoma (HCC) (3, 4). This condition not only increases the risk of serious liver-related complications but also imposes a substantial economic burden, costing the United States billions of dollars annually in healthcare expenditures and lost productivity (5).

Sedentary behavior and non-vigorous-activity have been identified as independent risk factors for MASLD, primarily through mechanisms involving metabolic dysregulation, insulin resistance, and increased hepatic fat accumulation (6–9). For instance, among obese adolescents, lower sedentary time is linked to reduced markers of fatty liver (10). However, the mechanisms by which prolonged sedentary behavior contributes to MASLD onset and progression remain insufficiently understood.

Inflammation plays a central role in MASLD pathogenesis. Various systemic inflammation and immune response markers—such as HSCRP, WBC, neutrophil-to-lymphocyte ratio (NLR), and platelet-to-albumin ratio (PAR)—reflect the underlying inflammatory state (11, 12). Although many studies have shown strong associations between MASLD and systemic inflammation (13–16). The precise immune mechanisms involved are still unclear. In particular, whether these inflammatory markers mediate the relationship between severe sedentary behavior and MASLD has not been thoroughly investigated.

Additionally, sedentary behavior is associated with elevated inflammatory markers, which may further exacerbate liver damage and contribute to MASLD pathogenesis (17–19). Understanding these relationships is crucial for developing effective public health strategies aimed at reducing sedentary time and addressing its detrimental effects on liver health. This study aims to investigate whether systemic inflammatory markers mediate the association between severe sedentary behavior and MASLD in overweight and obese individuals.

## 2 Methods

### 2.1 Study population

This cross-sectional study utilized data from the 2017–March 2020 pre-pandemic cycle of the NHANES, a nationally representative survey of the U. S. civilian non-institutionalized population conducted by the National Center for Health Statistics (NCHS). NHANES employs a complex, multistage probability sampling design to ensure representativeness.

**Figure 1:**
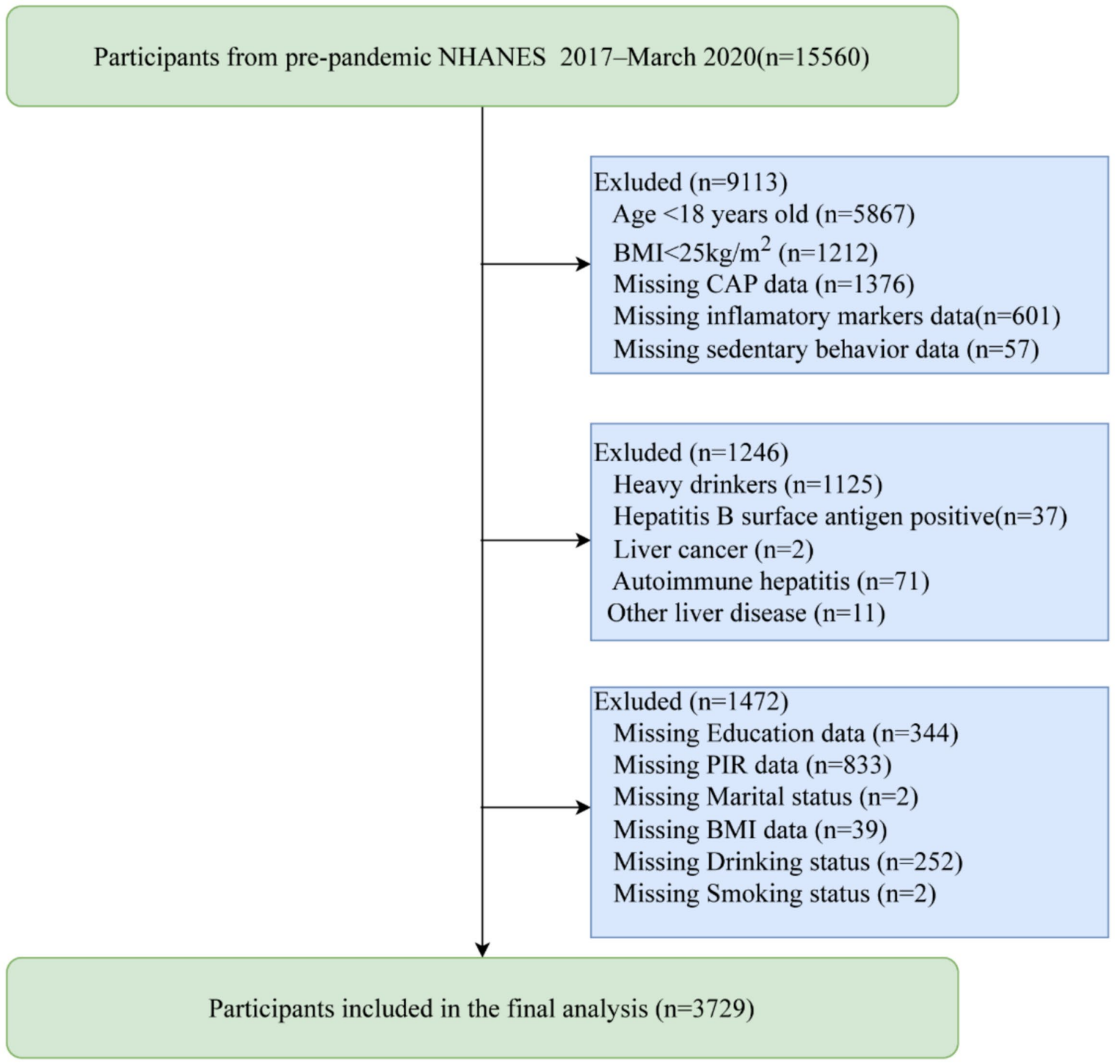
Flow chart for participants recruitment, NHANES 2017–2020.

We initially identified 15,560 participants from the NHANES program, all of whom voluntarily consented to participate in the study. We excluded individuals under 18 years of age (*n* = 5,867) and those with a BMI < 25 kg/m^2^ (*n* = 1,212). Participants with missing data on CAP (*n* = 1,376), inflammatory markers (*n* = 601), sedentary behavior (*n* = 57), or covariates (*n* = 1,472; education data (*n* = 344), family poverty income ratio (PIR) data (*n* = 833), marital status data (*n* = 2), BMI data (*n* = 39), drinking status data (*n* = 252),smoking status data (*n* = 2)) were also excluded from the analysis. Additionally, individuals with excessive alcohol consumption (*n* = 1,125) or other liver diseases (*n* = 121; including hepatitis B (*n* = 37), liver cancer (*n* = 2), autoimmune hepatitis (*n* = 71), and other liver diseases (*n* = 11)) were excluded. Ultimately, our study included 3,729 participants with a BMI ≥ 25 kg/m^2^, as shown in Figure 1.

### 2.2 Measures

#### 2.2.1 Definition of hepatic steatosis and its severity

The Controlled Attenuation Parameter (CAP) values were employed to define hepatic steatosis, categorizing it as follows: S1 for ≥5% steatosis (CAP ≥302 dB/m), S2 for ≥34% steatosis (CAP ≥331 dB/m), and S3 for ≥67% steatosis (CAP ≥337 dB/m). This methodology was validated through a multicenter prospective cross-sectional study conducted by Eddowes et al. (20), which demonstrated that a CAP cutoff of 302 dB/m exhibited robust diagnostic performance, achieving an area under the curve (AUC) of 0.87, with a sensitivity of 0.80 and a specificity of 0.83 for diagnosing S1. Furthermore, a cutoff value of 331 dB/m for CAP yielded an AUC of 0.77, sensitivity of 0.70, and specificity of 0.76 for diagnosing S2; while a cutoff of 337 dB/m for CAP provided an AUC of 0.70, sensitivity of 0.72, and specificity of 0.63 for diagnosing S3. Notably, these thresholds are consistent with the cutoff of 297 dB/m derived from a meta-analysis by Petroff et al. (21), further reinforcing their reliability. To ensure diagnostic accuracy, we adopted a CAP cutoff of ≥302 dB/m for the identification of hepatic steatosis.

#### 2.2.2 MASLD definition

MASLD was defined according to the American Association for the Study of Liver Diseases (AASLD) Practice Guidance on the clinical assessment and management of non-alcoholic fatty liver disease (1). Specifically, individuals with CAP ≥302 dB/m were classified as having MASLD if they met at least one of the following cardiometabolic risk factors:

- BMI ≥ 25 kg/m^2^ (or ≥23 kg/m^2^ for Asian populations), waist circumference >94 cm (men) or >80 cm (women), or ethnicity-adjusted equivalents;
- Fasting glucose ≥5.6 mmol/L (100 mg/dL), 2-h postprandial glucose ≥7.8 mmol/L (140 mg/dL), HbA1c ≥ 5.7% (39 mmol/mol), confirmed type 2 diabetes, or current antidiabetic therapy;
- Blood pressure ≥130/85 mmHg or antihypertensive treatment.
- Plasma triglycerides ≥1.70 mmol/L (150 mg/dL) or lipid-lowering therapy.
- Plasma HDL-cholesterol ≤1.0 mmol/L (40 mg/dL) in men or ≤1.3 mmol/L (50 mg/dL) in women, or lipid-lowering therapy.

#### 2.2.3 Exposure: sedentary behavior

Sedentary behavior was evaluated through a self-reported question: “How much time do you usually spend sitting on a typical day?” Participants were instructed to include all sitting time, excluding sleep. Those reporting ≥480 min/day of sitting were classified as having severe sedentary behavior, and those reporting <480 min/day were classified as having mild sedentary behavior (22, 23).

The threshold of 480 min/day aligns with existing epidemiological evidence and public health recommendations. Prolonged sedentary time—defined as sitting ≥8 h/day—is associated with elevated risks of adverse health outcomes, including cardiovascular diseases, type 2 diabetes, and all-cause mortality (24, 25). For instance, the American Heart Association emphasizes the importance of reducing sedentary time to improve metabolic health (25). This cut-off enables the identification of populations at high risk, facilitating targeted interventions.

#### 2.2.4 Measurement of inflammatory markers

All inflammatory markers were measured following standardized NHANES laboratory protocols. Peripheral blood samples were collected at the NHANES Mobile Examination Center (MEC). Automated cell counters (Beckman Coulter) were used to quantify white blood cells (WBC), neutrophils (NEU), lymphocytes, and platelets.

Albumin (ALB) levels, obtained from the standard biochemical module, were measured using the bromcresol purple (BCP) dye-binding method. High-sensitivity C-reactive protein (HSCRP), an acute-phase protein, was quantified via an immunoturbidimetric assay, providing sensitive detection of low-grade inflammation.

#### 2.2.5 Covariates

Covariates included age (years), sex (male/female), race/ethnicity (Non-Hispanic White, Non-Hispanic Black, Other Multiracial, Mexican American, Other Hispanic), education level (below high school, high school, above high school), marital status (Married/Living with Partner, Never Married, Widowed/Divorced/Separated), family poverty income ratio (PIR), smoking status, alcohol consumption, vigorous recreational activity, hypertension, diabetes, and dyslipidemia.

Smoking status was classified based on lifetime cigarette use and current smoking behavior, categorizing participants as never smokers (no lifetime smoking), former smokers (having smoked at least 100 cigarettes but not currently smoking), or current smokers. Drinking status was recorded as a binary variable (yes/no), with excessive drinking defined as a daily intake exceeding 20 g for men or 10 g for women, as determined from 24-h dietary recall data. Caloric intake (KCAL) was also determined from 24-h dietary recall data.

Vigorous recreational activity was assessed through participants’ self-reported levels of intense physical exercise. Diabetes was diagnosed if any of the following criteria were met: fasting blood glucose > 7.1 mmol/L, HbA1c > 6.5%, a physician’s diagnosis of diabetes, or current insulin treatment. Hypertension was defined as a positive response to having ever been diagnosed with high blood pressure by a doctor or health professional. Dyslipidemia was determined by meeting any of the following criteria: total cholesterol > 200 mg/dL, LDL ≥ 130 mg/dL, or HDL < 40 mg/dL in men and <50 mg/dL in women. These definitions were based on NHANES questionnaire data, laboratory measures, and standard clinical guidelines.

### 2.3 Statistical analysis

All analyses accounted for the complex, multistage sampling design of NHANES by using the survey package in R (version 4.3.3) with appropriate sampling weights (Full Sample MEC Exam weight) to ensure nationally representative estimates. Continuous variables are presented as medians (Q1, Q3), and categorical variables as counts (percentages). Group comparisons between MASLD and non-MASLD, as well as between severe and mild sedentary behavior groups, were conducted using the Wilcoxon rank-sum test for continuous variables and the chi-squared test with Rao and Scott’s second-order correction for categorical variables.

We excluded data with missing covariates, thereby employing complete case analysis for covariates in the multivariable models. Weighted multivariable logistic regression was utilized to evaluate the association between sedentary behavior and MASLD in participants with a BMI ≥ 25 kg/m^2^. In the crude model, no covariates were adjusted. Model 1 adjusted for age, sex, race/ethnicity, education, marital status, PIR, smoking status, and drinking status; Model 2 additionally included vigorous recreational activity, diabetes, hypertension, dyslipidemia and caloric intake. Weighted logistic regression was also applied to examine the association between inflammatory markers and MASLD (adjusted for all covariates), and weighted linear regression was used to assess the relationship between sedentary behavior and inflammatory markers.

Mediation analysis was conducted using the mediate function from the “mediation” package in R. To enhance the stability and accuracy of the results, we increased the number of simulations to 1,000. The direct effect represents the association between sedentary behavior and MASLD, the indirect effect is the portion mediated by inflammatory markers, and the proportion mediated indicates the relative contribution of this indirect pathway. All analyses were conducted in R (version 4.3.3, http://www.R-project.org). A two-sided *p*-value <0.05 was considered statistically significant.

## 3 Results

### 3.1 Baseline characteristics

Table 1 shows the basic demographic characteristics of the participants. This study included 3,729 participants (BMI ≥ 25 kg/m^2^), and 1,418 of whom with MASLD and 2,311 of whom without MASLD, with a weighted prevalence of 38.76%. Individuals with MASLD have a significantly longer sedentary duration (360 min) compared to those without MASLD (300 min), and they also exhibit a higher proportion of individuals not engaging in vigorous physical activities (*p* < 0.05). The median age was 53 years (IQR: 38–65), males accounted for 49.13% of participants, and the weighted prevalence of MASLD was higher in males than in females (43.44% vs. 34.05%, *p* < 0.001).

**Table 1:** Weighted baseline characteristics of the study population by MASLD status, NHANES 2017–March 2020.

Characteristic	All participants, *N* = 3729^1^	Non-MASLD, *N* = 2,311 (61%)^1^	MASLD, *N* = 1,418 (39%)^1^	*p*-value^2^
Age (year)	53.0 (38.0, 65.0)	48.0 (35.0, 63.0)	53.0 (38.0, 63.0)	**0.047**
Sex				**0.003**
Female	1,897 (50.87%)	1,284 (53.61%)	613 (43.72%)	
Male	1,832 (49.13%)	1,027 (46.39%)	805 (56.28%)	
Race				**0.002**
Non-Hispanic White	1,335 (35.80%)	771 (61.53%)	564 (65.79%)	
Non-Hispanic Black	976 (26.17%)	693 (13.31%)	283 (7.64%)	
Other/multiracial	509 (13.65%)	312 (8.37%)	197 (8.59%)	
Mexican American	491 (13.17%)	261 (8.30%)	230 (11.04%)	
Other Hispanic	418 (11.21%)	274 (8.49%)	144 (6.95%)	
Marital status				**0.009**
Married/Living with Partner	2,241 (60.10%)	1,337 (62.86%)	904 (69.59%)	
Never married	637 (17.08%)	432 (17.70%)	205 (14.29%)	
Widowed/Divorced/Separated	851 (22.82%)	542 (19.44%)	309 (16.12%)	
Education level				0.3
Below high school	656 (17.59%)	393 (9.99%)	263 (10.64%)	
High school	906 (24.30%)	539 (27.09%)	367 (30.39%)	
More than high school	2,167 (58.11%)	1,379 (62.92%)	788 (58.97%)	
PIR				0.8
<1	690 (18.50%)	428 (12.53%)	262 (12.53%)	
1–3	1,634 (43.82%)	1,016 (35.14%)	618 (36.10%)	
≥3	1,405 (37.68%)	867 (52.33%)	538 (51.36%)	
Smoking status				**0.006**
Never smoker	2,216 (59.43%)	1,441 (61.75%)	775 (56.73%)	
Former smoker	969 (25.99%)	527 (24.07%)	442 (31.53%)	
Current smoker	544 (14.59%)	343 (14.18%)	201 (11.74%)	
Drinking status				0.067
Non-drinker	341 (9.14%)	213 (6.27%)	128 (8.96%)	
Former/Current drinker	3,388 (90.86%)	2,098 (93.73%)	1,290 (91.04%)	
Vigorous recreational Activity				**<0.001**
No	2,930 (78.57%)	1,737 (68.88%)	1,193 (82.69%)	
Yes	799 (21.43%)	574 (31.12%)	225 (17.31%)	
Diabetes				**<0.001**
No	2,834 (76.00%)	1,946 (88.96%)	888 (68.59%)	
Yes	895 (24.00%)	365 (11.04%)	530 (31.41%)	
Hypertension				**<0.001**
No	2,116 (56.74%)	1,432 (69.24%)	684 (50.66%)	
Yes	1,613 (43.26%)	879 (30.76%)	734 (49.34%)	
Dyslipidemia				**<0.001**
No	1,471 (39.45%)	1,023 (43.45%)	448 (28.84%)	
Yes	2,258 (60.55%)	1,288 (56.55%)	970 (71.16%)	
Caloric intake (KCAL)	1,904.99 (1,456.50, 2,485.00)	1,865.39 (1,438.34, 2,397.50)	1,979.99 (1,510.67, 2,595.31)	**0.001**
Minutes sedentary activity (Minutes)	300 (180, 480)	300 (180, 480)	360 (240, 540)	**<0.001**
Sedentary behavior				**0.002**
Mild	2,632 (70.58%)	1,684 (70.45%)	948 (62.88%)	
Severe	1,097 (29.42%)	627 (29.55%)	470 (37.12%)	
WBC (10^9^/L)	7.10 (5.90, 8.60)	7.00 (5.90, 8.40)	7.70 (6.50, 9.30)	**<0.001**
NE (10^9^/L)	4.10 (3.10, 5.20)	4.00 (3.20, 5.00)	4.50 (3.60, 5.60)	**<0.001**
Albumin (g/dL)	4.00 (3.80, 4.20)	4.10 (3.90, 4.30)	4.10 (3.80, 4.20)	**0.004**
HSCRP (mg/L)	2.54 (1.15, 5.46)	1.98 (0.95, 4.07)	3.38 (1.72, 6.96)	**<0.001**

Sedentary behavior was classified as mild (<480 min/day) or severe (≥480 min/day). MASLD was defined as CAP ≥302 dB/m. PIR, poverty income ratio; WBC, white blood cell count; NE, neutrophil count; ALB, albumin; HSCRP, high-sensitivity C-reactive protein.

^1^Values are presented as median (Q1, Q3) for continuous variables and n (unweighted %) for categorical variables.

^2^*p*-values were derived using the Wilcoxon rank-sum test for continuous variables and the chi-square test with Rao and Scott’s second-order correction for categorical variables.

The bolded *p* value indicates that the difference is statistically significant.

Individuals with MASLD exhibited significantly higher HSCRP, WBC, and NE levels than those without MASLD. MASLD prevalence varied significantly by sex, race/ethnicity, marital status, smoking status, vigorous physical activity, and sedentary behavior (*p* < 0.05, Table 1). Among participants categorized by sedentary behavior, those in the severe sedentary group had a higher MASLD prevalence than those with mild sedentary behavior (44.29% vs. 36.10%, *p* = 0.002). Additionally, severe sedentary individuals exhibited elevated HSCRP levels (2.97 vs. 2.25, Supplementary Table S1).

### 3.2 The association between sedentary behavior, vigorous physical recreational activities, inflammatory markers, and MASLD at different threshold of CAP

In the unadjusted model, participants with severe sedentary behavior exhibited significantly higher odds of MASLD compared to those with mild sedentary behavior (OR = 1.41, 95% CI: 1.15–1.72; *p* = 0.002) at a 302 dB/m threshold. This association persisted after controlling for age, sex, race/ethnicity, education level, marital status, PIR, smoking status, and drinking status (Model 1), and remained stable following additional adjustments for vigorous recreational activity, diabetes, hypertension, dyslipidemia, and caloric intake (Model 2; OR = 1.43, 95% CI: 1.02–1.99; *p* = 0.041, Table 2).

**Table 2:** The association between sedentary behavior, vigorous physical recreational activities, grades of obesity, inflammatory markers, and MASLD at different threshold of CAP.

Factors	Cutoff value of CAP (302 dB/m)						Cutoff value of CAP (274 dB/m)					
	Crude model^a^		Model 1^b^		Model 2^c^		Crude model^a^		Model 1^b^		Model 2^c^	
	OR (95CI%)	*P*-value	OR (95CI%)	*P*-value	OR (95CI%)	*P*-value	OR (95CI%)	*P*-value	OR (95CI%)	*P*-value	OR (95CI%)	*P*-value
Types of SB												
Mild Sedentary behavior	Reference		Reference		Reference		Reference		Reference		Reference	
Severe Sedentary behavior	1.41(1.15, 1.72)	**0.002**	1.55(1.22, 1.96)	**0.002**	1.43(1.02, 1.99)	**0.041**	1.29(1.04, 1.59)	**0.022**	1.42(1.12, 1.81)	**0.009**	1.31(0.94, 1.82)	0.087
Types of SB and VRA												
Mild SB + VRA	Reference		Reference		Reference		Reference		Reference		Reference	
Mild SB + Non-VRA	2.00(1.47, 2.73)	**<0.001**	2.18(1.51, 3.15)	**0.002**	1.89(1.13, 3.16)	**0.029**	2.00(1.59, 2.52)	**<0.001**	2.11(1.59, 2.81)	**<0.001**	1.87(1.22, 2.85)	**0.018**
Severe SB + VRA	1.18(0.64, 2.20)	0.578	1.24(0.62, 2.49)	0.489	1.13(0.42, 3.03)	0.715	1.25(0.77, 2.05)	0.578	1.34(0.80, 2.26)	0.219	1.25(0.60, 2.59)	0.407
Severe SB + Non-VRA	3.02(2.28, 3.99)	**<0.001**	3.53(2.53, 4.94)	**<0.001**	2.88(1.77, 4.71)	**0.006**	2.65(2.15, 3.28)	**<0.001**	3.01(2.29, 3.95)	**<0.001**	2.49(1.62, 3.82)	**0.007**
Grades of obesity												
(25, 30) (kg/m^2^)	Reference		Reference		Reference		Reference		Reference		Reference	
≥30 (kg/m^2^)	4.17(3.34, 5.20)	**<0.001**	4.91(3.71, 6.48)	**<0.001**	3.93(2.69, 5.75)	**0.001**	4.17(3.34, 5.20)	**<0.001**	5.07(3.90, 6.60)	**<0.001**	4.23(2.83, 6.31)	**0.001**
Inflammatory markers												
HSCRP	1.05(1.01, 1.09)	**0.016**	1.06(1.01, 1.11)	**0.023**	1.04(0.99, 1.10)	0.10	1.04(1.00, 1.09)	0.068	1.05(0.99, 1.12)	0.074	1.03(0.97, 1.10)	0.200
ALB	0.60(0.44, 0.82)	**0.003**	0.45(0.32, 0.63)	**<0.001**	0.55(0.34, 0.88)	**0.024**	0.75(0.49, 1.15)	0.200	0.63(0.41, 0.98)	**0.044**	0.83(0.46, 1.48)	0.400
WBC	1.20(1.14, 1.26)	**<0.001**	1.23(1.15,1.31)	**<0.001**	1.16(1.07,1.27)	**0.009**	1.16(1.09, 1.22)	**<0.001**	1.17(1.10,1.25)	**<0.001**	1.12(1.02, 1.23)	**0.030**
NE	1.23(1.16,1.30)	**<0.001**	1.24(1.15, 1.34)	**<0.001**	1.17(1.04,1.31)	**0.019**	1.15(1.08, 1.24)	**<0.001**	1.15(1.06, 1.26)	**0.004**	1.09(0.96, 1.24)	0.140

OR, odds ratio; CI, confidence intervals; SB, sedentary behavior; VRA, vigorous recreational activity; WBC, white blood cell count; NE, neutrophil count; ALB, albumin; HSCRP, high-sensitivity C-reactive protein.

^a^Crude model: no covariates were adjusted.

^b^Model 1: age, sex, race, education, marital status, poverty income ratio, smoking status, and alcohol use status were adjusted.

^c^Model 2: age, sex, race, education, marital status, poverty income ratio, smoking status, alcohol use status, vigorous recreational activity, diabetes, hypertension, dyslipidemia, and caloric intake were adjusted.

The bolded *p* value indicates that the difference is statistically significant.

To investigate the relationship between sedentary behavior, vigorous physical activity types, inflammatory markers, and MASLD at a CAP threshold of 274 dB/m, we conducted a sensitivity analysis using alternative CAP cutoffs (e.g., 274 dB/m). The results indicated that in both the unadjusted model and the partially adjusted model, sedentary behavior remained significantly associated with MASLD (OR = 1.29, 95% CI: 1.04–1.59; *p* = 0.022; OR = 1.42, 95% CI: 1.12–1.81; *p* = 0.009). However, after adjusting for all covariates, the association between sedentary behavior and MASLD was no longer significant (OR = 1.31, 95% CI: 0.94, 1.82; *p* = 0.087) (Table 2).

When participants were categorized based on their sedentary status and levels of vigorous recreational activity, those classified as severely sedentary with minimal vigorous activity exhibited significantly higher odds of developing MASLD (OR = 2.88, 95% CI: 1.77–4.71; *p* = 0.006). Additionally, individuals in the mild sedentary and non-vigorous activity group also demonstrated a substantial increase in odds (OR = 1.89, 95% CI: 1.13–3.16; *p* = 0.029). This relationship remained significant under a CAP threshold of 274 dB/m (Table 2).

Weighted logistic regression analyses revealed that while albumin (ALB) exhibited a protective association with MASLD (OR = 0.55, 95% CI: 0.34–0.88; *p* = 0.024), both WBC (OR = 1.16, 95% CI: 1.07–1.27; *p* = 0.009) and NE (OR = 1.17, 95% CI: 1.04–1.31; *p* = 0.019) demonstrated positive associations across all three models. However, high-sensitivity C-reactive protein (HSCRP) did not reach statistical significance in model 2 (OR = 1.04, *p* = 0.10, Table 2). Under the threshold of CAP ≥ 274 dB/m, only WBC showed a significant correlation with MASLD across the three weighted logistic regression models, while the associations of other inflammatory markers were not significant (Table 2).

### 3.3 Relationship between inflammatory markers and different grades of hepatic steatosis

Based on the study by Eddowes et al. (20), we classified the stages of hepatic steatosis using the Controlled Attenuation Parameter (CAP) values as follows: S0 for CAP < 302 dB/m (<5% steatosis), S1 for CAP ≥ 302 dB/m and <331 dB/m (≥5% steatosis), S2 for CAP ≥ 331 dB/m and <337 dB/m (≥34% steatosis), and S3 for CAP ≥ 337 dB/m (≥67% steatosis). We analyzed the differences in inflammatory marker levels across these stages of hepatic steatosis. The results indicated statistically significant differences in the concentrations of HSCRP, WBC, NE, and ALB among the various degrees of hepatic steatosis, with all *p*-values being less than 0.05. Notably, the levels of WBC and NE increased progressively from S0 to S3 (Table 3).

**Table 3:** The comparison of inflammatory marker levels among different grades of hepatic steatosis.

Inflammatory markers	S0 Median (M1, M3)	S1 Median (M1, M3)	S2 Median (M1, M3)	S3 Median (M1, M3)	*P*-value
HSCRP (mg/L)	2.10(0.97,4.42)	2.93(1.42,6.23)	2.76(1.47,5.62)	3.95(2.00,7.67)	<0.001
WBC (10^9^/L)	6.80(5.70,8.30)	7.30(6.10,8.60)	7.50(6.10,9.00)	7.80(6.50,9.30)	<0.001
NE (10^9^/L)	3.90(3.00,4.90)	4.20(3.30,5.30)	4.30(3.27,5.80)	4.50(3.60,5.60)	<0.001
ALB (g/dL)	4.00(3.80,4.30)	4.10(3.80,4.30)	4.10(3.80,4.30)	4.00(3.80,4.20)	0.009

WBC, white blood cell count; NE, neutrophil count; ALB, albumin; HSCRP, high-sensitivity C-reactive protein. S0 (<5% steatosis); S1 (≥5% steatosis); S2 (≥34% steatosis); S3 (≥67% steatosis).

### 3.4 The correlation between different obesity grades and sedentary behaviors and MASLD, inflammatory markers

We analyzed the correlations between different levels of obesity, sedentary behavior, inflammatory markers AND MASLD. Our results indicated that individuals with a BMI of ≥30 kg/m^2^ exhibited a significant association with MASLD compared to those in the BMI range of (25, 30) kg/m^2^ across three weighted logistic regression models, with all p values being less than 0.05 (Table 2). Furthermore, our analysis revealed a significant correlation between inflammatory markers and different levels of obesity; specifically, individuals in the BMI ≥ 30 kg/m^2^ group had higher levels of HSCRP, WBC, and NE compared to those in the BMI (25, 30) kg/m^2^ group, while ALB levels were lower in the former group (Supplementary Table S2).

### 3.5 Subgroup analysis

**Figure 2:**
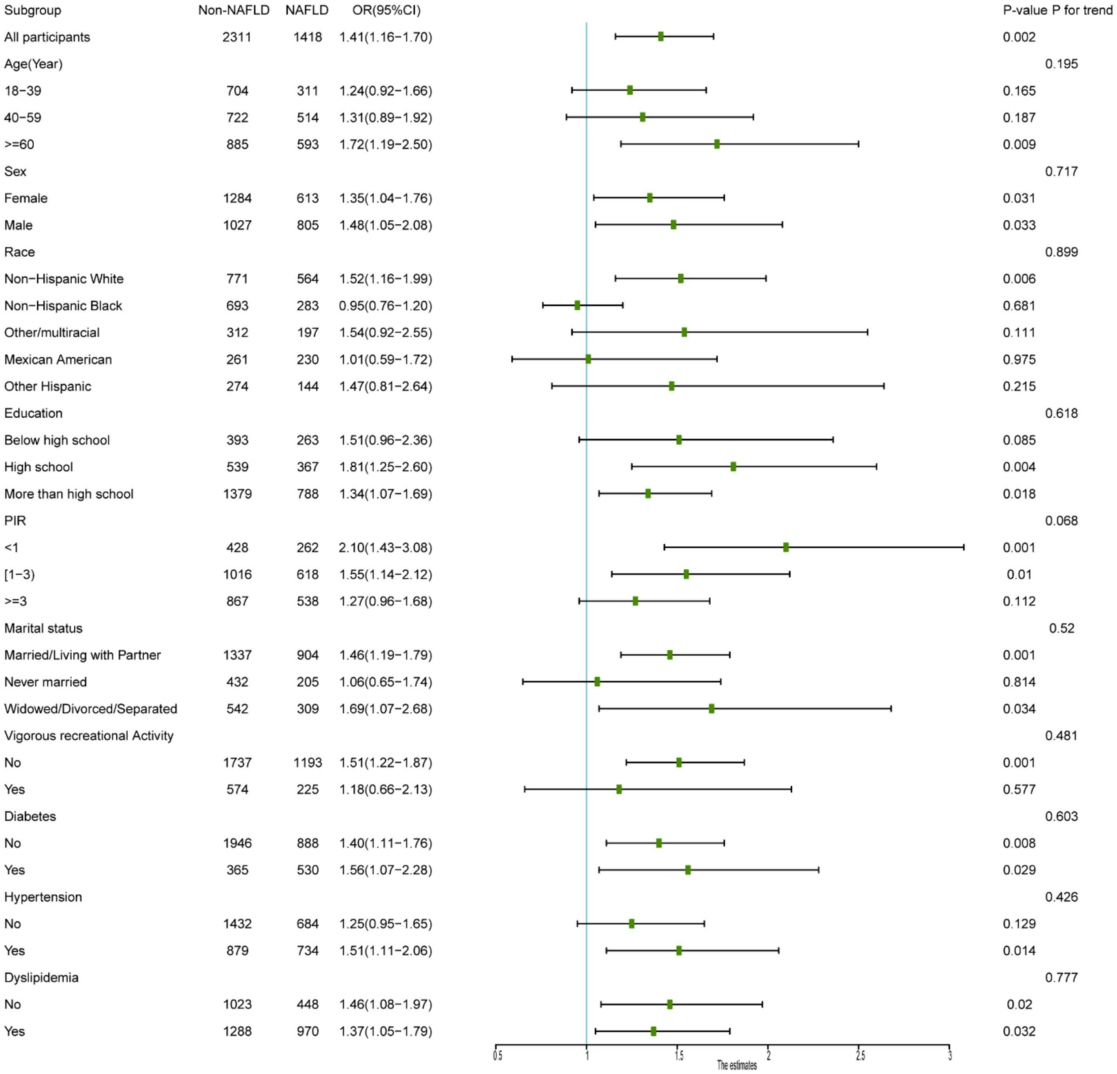
Subgroup analysis forest plot of sedentary behavior and MASLD in different subgroups.

As shown in Figure 2, subgroup analyses indicated that the heightened MASLD risk associated with severe sedentary behavior extended across a diverse range of populations, including older adults (≥60 years), Non-Hispanic White individuals, with higher education levels, those with lower household income, married participants, those without vigorous recreational activities, and individuals with hypertension. That severe sedentary behavior emerges as a common risk factor across multiple demographic and health strata underscores its pervasive influence, suggesting that public health interventions aimed at reducing sedentary time could yield widespread metabolic health benefits.

### 3.6 The association between sedentary behavior and inflammatory markers

After adjusting for multiple covariates, severe sedentary behavior was strongly associated with increased HSCRP (*β* = 0.92), WBC (*β* = 0.19), and NE (*β* = 0.11) levels, and decreased ALB (*β* = −0.04) levels (all *p* < 0.05, Supplementary Table S3). These alterations in inflammatory and nutritional markers suggest that a state of heightened inflammation and diminished hepatic synthetic function may link sedentary behavior to MASLD pathogenesis. This aligns with the broader understanding that prolonged inactivity not only alters metabolic profiles but also fosters an inflammatory milieu that could accelerate liver damage.

### 3.7 Mediation analysis

**Figure 3:**
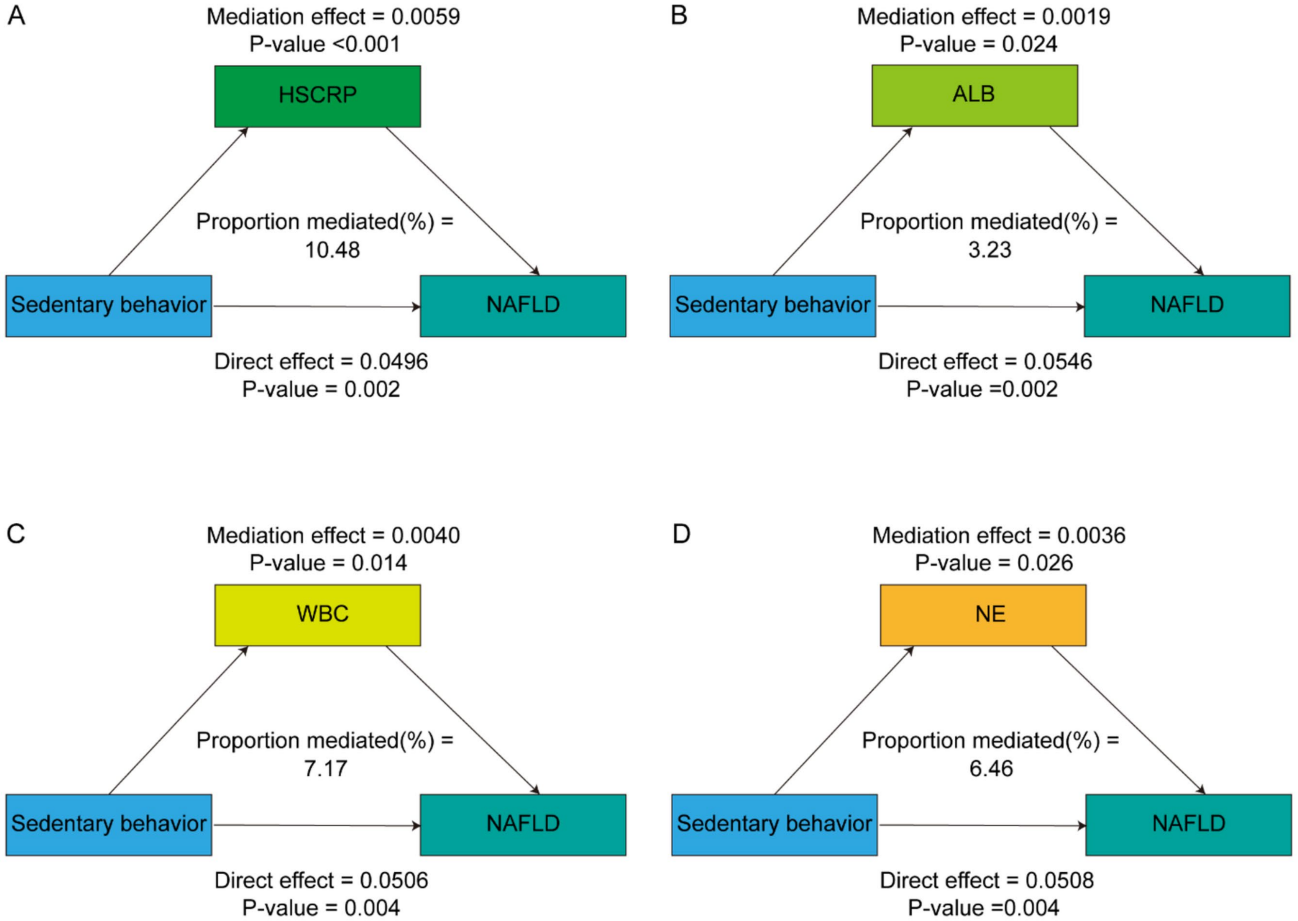
Path diagram of the mediation analysis of inflammatory biomarkers on the relationship between sedentary behavior and MASLD. The graphs in **(A–D)** represented the mediating role of HSCRP, ALB, WBC and NE, respectively.

After comprehensive adjustment for demographic, socioeconomic, lifestyle, and clinical covariates, HSCRP, ALB, WBC and NE significantly mediated the SB – MASLD relationship, accounting for 10.48, 3.23, 7.17, and 6.46% of the association, respectively (all *p* < 0.05) (Table 4 and Figure 3). These mediation results strengthen the argument that inflammation and related biological pathways represent key mechanistic links between sedentary lifestyles and MASLD risk. Identifying and targeting these pathways could provide more precise strategies for preventing or slowing the disease’s progression.

**Table 4:** The mediation effects of inflammatory markers.

Inflammatory markers	Proportion of mediated (average)	95%CI	*P*-value
HSCRP	0.1048	0.0382–0.2700	<0.001
ALB	0.0323	0.0035–0.1100	0.024
WBC	0.0717	0.0105–0.2300	0.014
NE	0.0646	0.0078–0.2000	0.026

WBC, white blood cell count; NE, neutrophil count; ALB, albumin; HSCRP, high-sensitivity C-reactive protein.

## 4 Discussion

This study provides new evidence that blood-cell-based inflammatory markers, HSCRP and ALB, may partially mediate the relationship between severe sedentary behavior and MASLD in a nationally representative sample of overweight and obese adults.

We confirmed that severe sedentary behavior remained significantly associated with MASLD after adjusting for multiple covariates. Moreover, elevated WBC and NEU counts were linked to MASLD, underscoring the role of a pro-inflammatory state in the disease process. Notably, our mediation analysis indicated that HSCRP, ALB, WBC and NE help explain how sedentary behavior contributes to MASLD risk.

These findings align with previous research showing that low-grade inflammation and altered immune responses play a central role in MASLD pathogenesis (14, 26, 27). Chronic metabolic disturbances—such as those stemming from obesity, adipose tissue dysfunction, and gut-liver axis impairment—promote lipid accumulation, endoplasmic reticulum stress, and recruitment of immune cells, ultimately triggering sustained inflammatory responses (28, 29). Our observation that MASLD is associated with increased WBC and NEU counts is consistent with the recognized importance of neutrophils and their mediators in the progression of fatty liver disease (30–32). Likewise, the inverse association between ALB and MASLD highlights how chronic liver inflammation impairs albumin synthesis and function, contributing to disease severity. Interestingly, although HSCRP has been implicated as a predictor of MASLD in prior studies (15, 16, 33, 34), its direct association was not significant in our fully adjusted models. This discrepancy may stem from variations in population characteristics, the stage of MASLD, or the specific CAP cutoff employed. We used a CAP threshold of ≥302 dB/m to define MASLD, whereas some studies recommend alternative cutoffs (e.g., ≥274 dB/m) (23). The selection of CAP cutoff values significantly influences MASLD prevalence estimates, as evidenced by our findings and existing literature. Our analysis revealed a prevalence of 38.76% at a 302 dB/m threshold, while lowering the cutoff to ≥274 dB/m substantially increased the weighted prevalence to 56.87% in overweight and obese population, reflecting enhanced diagnostic sensitivity at the cost of reduced specificity—a trade-off that amplifies risks of overestimation in population-level assessments. These observations contrast with the 31.9% prevalence reported by Kim et al. (35) using a 285 dB/m threshold, underscoring the critical variability introduced by diagnostic criteria. Collectively, these disparities highlight the urgent need for standardized CAP thresholds to ensure epidemiological accuracy, mitigate overdiagnosis biases, and reconcile sensitivity-specificity imbalances in MASLD research. Nonetheless, the significant mediating role of HSCRP suggests that inflammation remains a crucial pathway linking sedentary behavior to MASLD.

Our results reinforce the notion that sedentary behavior, in addition to its well-documented metabolic and cardiovascular consequences (6, 7, 9, 36–38), is significantly associated with MASLD. Interestingly, when the cutoff value for defining MASLD via CAP is lowered from 302 dB/m to 274 dB/m, the association between severe sedentary behavior and MASLD becomes non-significant. This attenuated relationship at CAP ≥ 274 dB/m may arise from reduced disease specificity. A higher cutoff (CAP ≥ 302 dB/m) likely identifies advanced steatosis with more pronounced metabolic disturbances that are sensitive to lifestyle factors, whereas lower thresholds may encompass a heterogeneous group of subclinical cases where the effects of sedentary behavior are diluted by other contributing factors (39, 40). This underscores the cutoff-dependent nature of relationships within the pathophysiology of MASLD (41).

Our study also revealed that the levels of inflammatory markers vary across different degrees of steatosis, with WBC and NE levels progressively increasing from S0 to S3. This variation in inflammation may influence the relationship between SB and MASLD. Consequently, the strength or presence of the SB-MASLD association may be sensitive to the definition or severity of steatosis. Furthermore, this sensitivity may reflect underlying pathophysiological mechanisms, as varying CAP thresholds capture different degrees of hepatic fat accumulation, which can affect inflammatory responses and metabolic dysregulation related to sedentary behavior.

Additionally, our analysis indicates a significant difference in MASLD prevalence between overweight or obese men (43.44%) and women (34.05%, *p* < 0.001). This disparity may be attributed to hormonal differences, as estrogen may protect against MASLD in women (42). Additionally, men tend to have more visceral fat and engage in less health-conscious behaviors, contributing to higher MASLD rates (43, 44). These findings highlight the need for targeted public health strategies for men, particularly those who are overweight or obese, to address the rising burden of MASLD.

The strengths of this study include its use of a large, nationally representative dataset and adjustment for a wide range of sociodemographic and lifestyle variables, thus enhancing the robustness and generalizability of our findings. Introducing blood-cell-based inflammatory markers as mediators of the sedentary behavior–MASLD relationship is a novel contribution that deepens our understanding of the underlying pathophysiology and may inform targeted interventions.

Several limitations must be acknowledged. The cross-sectional design restricts causal inferences. Although the mediation effect of ALB was statistically significant, its modest contribution of only 3.23% may reflect its role as a compensatory anti-inflammatory protein rather than a direct mediator of inflammation related to MASLD. Other inflammatory markers, such as HSCRP and WBC, likely exert a stronger influence on the relationship between sedentary behavior and MASLD (45–47). Thus, while statistically significant, the practical significance of ALB’s mediation effect warrants further investigation. Future longitudinal studies are warranted to confirm our mediation findings. Use of ultrasound transient elastography and reliance on a single CAP threshold (≥302 dB/m) may influence diagnostic accuracy and comparability with studies using different cutoffs. Additionally, the timing of blood sample collection relative to survey data and the lack of information on certain clinical conditions (e.g., medication use, COPD) may have introduced residual confounding. Further research should incorporate more refined MASLD diagnostic techniques, assess additional covariates, and investigate potential biological mechanisms that were not directly measured in this study.

In summary, our data suggest that systemic inflammation—reflected by key markers—partially mediates the relationship between severe sedentary behavior and MASLD prevalence. These findings highlight the importance of reducing sedentary habits and addressing underlying inflammation in overweight and obese individuals. Future longitudinal studies are needed to determine if decreasing sedentary time and increasing vigorous recreational activity can lower the risk of MASLD.

## Data Availability

The datasets presented in this study can be found in online repositories. The names of the repository/repositories and accession number(s) can be found: https://www.cdc.gov/nchs/nhanes/.
