# Supplementary material for: The association between sedentary behavior and MASLD in overweight and obese adults: investigating the role of inflammatory markers using NHANES data (2017–March 2020)
Source: Front Nutr. 2025 Jun 27;12:1579453. doi: 10.3389/fnut.2025.1579453 (PMC12246849; doi:10.3389/fnut.2025.1579453)
Supplement: Supplementary file 1 [file Supplementary_file_1.DOCX]

Supplementary tables

Table S1 Weighted Baseline Characteristics of the Study Population by **sedentary** behavior

| **Characteristic** | **All participants**,  **N = 3,729**^1^ | **Mild sedentary**,  N = 2632 (68%)^2^ | **Severe sedentary**,  N = 1097 (32%)^2^ | **p-value**^3^ |
| --- | --- | --- | --- | --- |
| **MASLD** |  |  |  | **0.002** |
| No | 2,311 (61.97%) | 1,684 (63.90%) | 627 (55.71%) |  |
| Yes | 1,418 (38.03%) | 948 (36.10%) | 470 (44.29%) |  |
| **Age(Year)** | 53.00 (38.00, 65.00) | 51.00 (35.00, 63.00) | 49.00 (37.00, 62.00) | 0.400 |
| **Sex** |  |  |  | 0.700 |
| Female | 1,897 (50.87%) | 1,335 (49.45%) | 562 (50.45%) |  |
| Male | 1,832 (49.13%) | 1,297 (50.55%) | 535 (49.55%) |  |
| **Race** |  |  |  | **<0.001** |
| Non-Hispanic White | 1,335 (35.80%) | 865 (60.18%) | 470 (69.40%) |  |
| Non-Hispanic Black | 976 (26.17%) | 686 (11.65%) | 290 (9.99%) |  |
| Other/multiracial | 509 (13.65%) | 330 (7.98%) | 179 (9.44%) |  |
| Mexican American | 491 (13.17%) | 411 (11.33%) | 80 (5.28%) |  |
| Other Hispanic | 418 (11.21%) | 340 (8.85%) | 78 (5.89%) |  |
| **Marital status** |  |  |  | 0.300 |
| Married/Living with Partner | 2,241 (60.10%) | 1,608 (66.46%) | 633 (63.41%) |  |
| Never married | 637 (17.08%) | 428 (15.56%) | 209 (18.07%) |  |
| Widowed/Divorced/Separated | 851 (22.82%) | 596 (17.98%) | 255 (18.52%) |  |
| **Education** |  |  |  | **<0.001** |
| Below high school | 656 (17.59%) | 550 (12.33%) | 106 (5.91%) |  |
| High school | 906 (24.30%) | 682 (31.64%) | 224 (21.57%) |  |
| More than high school | 2,167 (58.11%) | 1,400 (56.03%) | 767 (72.52%) |  |
| **PIR** |  |  |  | **<0.001** |
| <1 | 690 (18.50%) | 541 (14.58%) | 149 (8.28%) |  |
| [1-3) | 1,634 (43.82%) | 1,196 (38.26%) | 438 (29.80%) |  |
| ≥3 | 1,405 (37.68%) | 895 (47.16%) | 510 (61.91%) |  |
| **Smoke** |  |  |  | 0.200 |
| Never smoker | 2,216 (59.43%) | 1,570 (58.45%) | 646 (62.62%) |  |
| Former smoker | 969 (25.99%) | 672 (27.38%) | 297 (26.09%) |  |
| Current smoker | 544 (14.59%) | 390 (14.17%) | 154 (11.29%) |  |
| **Drinking status** |  |  |  | 0.150 |
| Non-drinker | 341 (9.14%) | 255 (7.91%) | 86 (6.08%) |  |
| Former/Current drinker | 3,388 (90.86%) | 2,377 (92.09%) | 1,011 (93.92%) |  |
| **Vigorous recreational Activity** |  |  |  | 0.600 |
| No | 2,930 (78.57%) | 2,066 (74.55%) | 864 (73.58%) |  |
| Yes | 799 (21.43%) | 566 (25.45%) | 233 (26.42%) |  |
| **Diabetes** |  |  |  | 0.700 |
| No | 2,834 (76.00%) | 2,010 (81.27%) | 824 (80.63%) |  |
| Yes | 895 (24.00%) | 622 (18.73%) | 273 (19.37%) |  |
| **Hypertension** |  |  |  | **0.032** |
| No | 2,116 (56.74%) | 1,535 (64.02%) | 581 (57.91%) |  |
| Yes | 1,613 (43.26%) | 1,097 (35.98%) | 516 (42.09%) |  |
| **Dyslipidemia** |  |  |  | 0.400 |
| No | 1,471 (39.45%) | 1,056 (38.46%) | 415 (36.39%) |  |
| Yes | 2,258 (60.55%) | 1,576 (61.54%) | 682 (63.61%) |  |
| **Caloric intake(KCAL)** | 1,904.99 (1,456.50, 2,485.00) | 1,901.95 (1,440.75, 2,493.51) | 1,915.11 (1,479.43, 2,455.56) | 0.800 |
| **WBC(10^9^/L)** | 7.10 (5.90, 8.60) | 7.20 (6.10, 8.60) | 7.40 (6.00, 8.80) | 0.120 |
| **NE(10^9^/L)** | 4.10 (3.10, 5.20) | 4.20 (3.30, 5.30) | 4.30 (3.40, 5.40) | 0.200 |
| **PLT(10^9^/L)** | 243.00 (206.00, 286.00) | 243.00 (209.00, 284.00) | 250.00 (213.62, 291.68) | 0.093 |
| **Albumin(g/dL)** | 4.00 (3.80, 4.20) | 4.10 (3.90, 4.30) | 4.10 (3.90, 4.30) | 0.300 |
| **HSCRP(mg/L)** | 2.54 (1.15, 5.46) | 2.25 (1.11, 4.64) | 2.97 (1.12, 5.94) | **0.005** |

Sedentary behavior was classified as mild (<480 min/day) or severe (≥480 min/day). MASLD was defined as CAP ≥302 dB/m. Abbreviations: PIR, Poverty Income Ratio; WBC, White Blood Cell count; NE, Neutrophil count; PLT, Platelet count; ALB, Albumin; HSCRP, High-Sensitivity C-Reactive Protein.

1n (%); Median (IQR)

2n (unweighted) (%); Median (IQR)

3chi-squared test with Rao & Scott's second-order correction; Wilcoxon rank-sum test for complex survey samples

Table S2 Comparison of Sedentary Behavior and Inflammatory Markers Across Different Grades of Obesity

| **Characteristic** | **Overall**, N = 3729^1^ | **BMI(**kg/m²**)**  **[25,30)**, N = 1534 (41%)^1^ | **BMI(kg/m²)**  **>=30**, N = 2195 (59%)^1^ | **p-value**^2^ |
| --- | --- | --- | --- | --- |
| **Sedentary behavior** |  |  |  | **<0.001** |
| Mild | 2,632 (67.52%) | 1,148 (73.24%) | 1,484 (63.59%) |  |
| Severe | 1,097 (32.48%) | 386 (26.76%) | 711 (36.41%) |  |
| **HSCRP(mg/L)** | 2.45 (1.11, 5.20) | 1.58 (0.79, 3.19) | 3.26 (1.64, 6.36) | **<0.001** |
| **WBC(10^9^/L)** | 7.30 (6.10, 8.70) | 6.80 (5.70, 8.40) | 7.50 (6.30, 8.90) | **<0.001** |
| **NE(10^9^/L)** | 4.20 (3.30, 5.30) | 4.00 (3.10, 5.06) | 4.40 (3.50, 5.50) | **<0.001** |
| **ALB(g/dL)** | 4.10 (3.90, 4.30) | 4.20 (3.90, 4.30) | 4.00 (3.80, 4.20) | **<0.001** |
| ^1^Median (IQR); n (unweighted) (%) | | | | |
| ^2^Wilcoxon rank-sum test for complex survey samples; chi-squared test with Rao & Scott's second-order correction | | | | |

TABLE S3 The associations between Sedentary behavior and inflammatory biomarkers

|  | HSCRP  β(95%CI,P-value | ALB  β(95%CI,P-value | WBC  β(95%CI,P-value | NE  β(95%CI,P-value |
| --- | --- | --- | --- | --- |
| **Sedentary_behavir** |  |  |  |  |
| Mild(Reference) | - | - | - | - |
| Severe | 0.92  (0.43-1.4, **<0.001)** | -0.04  (-0.06, -0.02, **<0.001)** | 0.19  (0.05-0.33, **0.008)** | 0.11  (0.00-0.22, **0.045)** |

Adjusted for age, sex, race, marital status, education attainment, poverty income ratio, smoking status, alcohol drinking status, physical activity status, diabetes, hypertension, dyslipidemia and caloric intake.

β, coefficient; CI, confidence intervals.
